# Whole-Body Cryotherapy in Athletes: From Therapy to Stimulation. An Updated Review of the Literature

**DOI:** 10.3389/fphys.2017.00258

**Published:** 2017-05-02

**Authors:** Giovanni Lombardi, Ewa Ziemann, Giuseppe Banfi

**Affiliations:** ^1^Laboratory of Experimental Biochemistry and Molecular Biology, I.R.C.C.S. Istituto Ortopedico GaleazziMilan, Italy; ^2^Department of Physiology and Pharmacology, Gdansk University of Physical Education and SportGdansk, Poland; ^3^Vita-Salute San Raffaele UniversityMilan, Italy

**Keywords:** whole-body cryotherapy, cryochamber, recovery, inflammation, metabolic effects, irisin

## Abstract

Nowadays, whole-body cryotherapy is a medical physical treatment widely used in sports medicine. Recovery from injuries (e.g., trauma, overuse) and after-season recovery are the main purposes for application. However, the most recent studies confirmed the anti-inflammatory, anti-analgesic, and anti-oxidant effects of this therapy by highlighting the underlying physiological responses. In addition to its therapeutic effects, whole-body cryotherapy has been demonstrated to be a preventive strategy against the deleterious effects of exercise-induced inflammation and soreness. Novel findings have stressed the importance of fat mass on cooling effectiveness and of the starting fitness level on the final result. Exposure to the cryotherapy somehow mimics exercise, since it affects myokines expression in an exercise-like fashion, thus opening another possible window on the therapeutic strategies for metabolic diseases such as obesity and type 2 diabetes. From a biochemical point of view, whole-body cryotherapy not always induces appreciable modifications, but the final clinical output (in terms of pain, soreness, stress, and post-exercise recovery) is very often improved compared to either the starting condition or the untreated matched group. Also, the number and the frequency of sessions that should be applied in order to obtain the best therapeutic results have been deeply investigated in the last years. In this article, we reviewed the most recent literature, from 2010 until present, in order to give the most updated insight into this therapeutic strategy, whose rapidly increasing use is not always based on scientific assumptions and safety standards.

## Introduction

Local and systemic cold therapies (cryotherapies) are widely used to relieve symptoms of various diseases including inflammation, pain, muscle spasms, and swelling, especially chronic inflammatory ones, injuries, and overuse symptoms (Bettoni et al., 2013; Jastrzabek et al., 2013). The beneficial effects of cold as a therapeutic agent have been known for a long time, with ancient population aware about the reinvigorating effects of cold water either taken orally or used for baths. The use of cold, mainly locally, still remains in our daily common activities. A still up-to-date survey of a sample of Irish emergency physicians highlighted the fact that 73% of consultants frequently “prescribe” cold, 7% never suggest to use cryotherapy, and 30% is unsure about the benefits of using cold. Experience (47%) and common sense (27%) were the most frequently declared reasons for using ice, while only 17% referred to scientific reasoning (Collins, 2008).

Forty years ago, following personal observations of Prof. Toshiro Yamauchi (who recognized that the combination of cold and physical exercise was beneficial for clinical outcomes of treatments received by his patients', affected by rheumatoid arthritis, coming back from mountain localities after winter holidays), whole-body cryotherapy was introduced into clinical practice (Yamauchi et al., 1981a,b).

At present, the use of very cold air in special, controlled chambers may be proposed for treating symptoms of various diseases (Bouzigon et al., 2016). Beside its clinical applications, a brief full body exposure to dry air at cryogenic temperatures lower than −110°C has become widely popular in sports medicine, often used to enhance recovery after injuries and to counteract inflammatory symptoms resulting from overuse or pathology (Furmanek et al., 2014). The number of studies about the use of whole-body cryotherapy (WBC) in sports medicine is growing, however, it is still lower than the topic's potential if the wide range of application of this methodology is considered. Studies published on athletes had mainly focused on post-training or competitive season recovery. Only a limited number of papers had investigated the effects of WBC used in preparation phase for competitive season to enhance form and performance, or during periods of high intensity of training to limit overuse and overreaching. Studies should be acknowledged to define safety, effectiveness, and efficacy of the treatment in athletes and to discover underlying molecular mechanisms supporting the claimed beneficial effects.

This review article collects the most recent literature (since 2010, Banfi et al., 2010b) on whole-body cryotherapy with the purpose of delivering a complete and updated overview of the newest findings and the directions taken in research in this field. In particular, given the high number of new scientific findings mostly associated with great technological developments of this therapeutic method, this review discusses both technical aspects (i.e., therapeutic protocols, contraindications, thermoregulatory responses) and effects on a wide range of physiological (i.e., hematological, metabolic, energetic, endocrinological, skeletal, muscular, inflammatory) and functional parameters (post-exercise and post-traumatic recovery, pain, performance). We are aware of the limitations of this literature review. Almost all published research included in this review discuss results of using whole-body cryotherapy without providing any insight into molecular mechanisms involved in observed responses to the treatment. Also, although the review takes a non-systematic approach, an alternative meta-analysis would only offer a limited article coverage due to the type and, sometimes, the quality of available papers. Furthermore, we only reviewed reports on the WBC procedures performed in cryochambers (regardless of the cooling system, but considering the operating temperature); we do not consider treatments performed in cryosauna (also named cryocabins). Exposure to cold in a cryosauna cannot be deemed whole-body since during the treatment the head remains outside of the cabin. The two settings were concluded to, activate different molecular pathways and, possibly, exert different outcomes. Indeed, in a cryosauna, cooling is delivered through direct insufflation of liquid nitrogen vapors into the box. Free vapors are heavy and tend to remain within the cabin, below the chin; contrarily, in a nitrogen-cooled cryochamber liquid nitrogen fluxes through pipes inside the chamber's wall, and thus, there is no free nitrogen within the chamber. These differences also account for different safety standards of these treatments: free nitrogen vapor in a cryosauna could be potentially hazardous due to the risk of asphyxia.

In the present paper we refer to “whole-body cryotherapy,” which is the most commonly used term to define the methodology, but also to “whole-body cryostimulation,” which better describes effects of WBC in improving the metabolic and inflammatory responses as well as in enhancing recovery from exercise and injuries. In contrast, the term “cryotherapy” refers to a real therapy aimed at treating painful symptoms of inflammatory or traumatic conditions.

## Technical aspects

### Standardized protocol for WBC

WBC is performed in special chambers, with the temperature and humidity strictly controlled. A subject, minimally dressed (for e.g., bathing suit, socks, clogs, headband, and surgical mask to avoid direct exhalation of humid air), enters a vestibule chamber at −60°C, where he stays for about 30 s of body adaptation and then passes to a cryochamber at −110° to −140°C, depending on the cooling system (electrical or nitrogen), where he remains for no more than 3 min. It is mandatory to remove any sweat before entry to avoid the risk of skin burning and necrosis. Access to the chamber is allowed only in the presence of a skilled personnel, controlling the procedures. A patients is free to leave the chamber at any time.

### Contraindications

Being a medical therapy, WBC should follow strict guidelines and indications. Currently accepted contraindications for WBC include: cryoglobulinaemia, cold intolerance, Raynaud disease, hypothyroidism, acute respiratory system disorders, cardio-vascular system diseases (unstable angina pectoris, cardiac failure in III and IV stage according to NYHA), purulent-gangrenous cutaneous lesions, sympathetic nervous system neuropathies, local blood flow disorders, cachexia, and hypothermia, as well as claustrophobia and mental disorders hindering cooperation with patients during the treatment. When performed in the appropriate and controlled conditions, WBC is a safe procedure, which was demonstrated to be deleterious neither for lung (Smolander et al., 2006) nor heart function (Banfi et al., 2009a); however, recorded observation of a very slight, clinically irrelevant increase in the systolic blood pressure (Lubkowska and Szygula, 2010) justifies precautions indicated for patients affected by cardiovascular conditions.

### Temperature changes

Studying body temperature modifications following WBC, in comparison to changes observed in response to other cooling techniques, represents a hot topic. This is thought to be important since cooling effectiveness is the function of temperature decrease within a certain range.

Shifts in skin temperature (T_sk_) of chosen body regions monitored by thermography and contact thermometry, before and immediately after a single WBC session (30 s at −60°C, 3 min at −120°C) showed, for the first time, the influence of body mass index (BMI) on the range of alternations. The highest magnitude of temperature changes was observed within lower extremities (tibias: −8.7°C; feet: −5.2°C), the mean total body temperature decreased by 5.8°C, while the internal body temperature dropped only by 0.8°C. The mean changes of temperatures at different sites correlated with BMI (*r* = −0.46); for example, explicative images show that temperature decreased down to 8.1° and 7.9°C in a thin volunteer (BMI <25 kg/m^2^) and down to 4.8° and 5.5°C in an obese participant (BMI > 30 kg/m^2^), in the chest and back regions, respectively (Cholewka et al., 2012). Even more precisely than BMI, the fat-free mass index (FFMI: fat-free mass/height^2^) and body fat percentage in males were both found to correlate with changes in skin temperature following WBC, (Hammond et al., 2014). Body composition was, thus, observed to be one of the main determinants of potential temperature changes and, possibly, of therapy's effectiveness. Cooling efficacy, indeed, differs between males and females as demonstrated by Hammond et al.; however, despite females having higher levels of adiposity than males, they experience greater mean temperature changes compared to males (12.07 ± 1.55°C vs. 10.12 ± 1.86°C). Compared to males, females have 20% smaller body mass, 14% more fat, 33% smaller lean body mass, and 18% smaller surface area, a higher subcutaneous to visceral fat ratio and a smaller ratio of fat mass index (FMI) to FFMI. Furthermore, females' BSA-to-mass ratio is higher than males, and the heat loss increases proportionally to this ratio. Under cold stress, females have a more extensively vasoconstricted periphery, with greater surface heat losses and show a significantly reduced sensitivity of the shivering response. Taken together these evidences could explain the discrepancy in cooling efficiency between sexes (Hammond et al., 2014).

Costello analyzed reduction in skin, muscle (vastus lateralis, at 1, 2, and 3 cm) and rectal temperatures following a single exposure to either WBC (−110°C) or cold-water immersion (CWI, at 8°C). Immediately after these procedures, the maximum drop in T_sk_ was observed with WBC (−12.1 ± 1.0°C), marking a bigger drop compared to CWI (−8.8 ± 2.0°C). On the contrary, core (−0.3° to −0.4°C) and muscle (−1.2° to −2.0°C) temperatures shifted slightly with no differences between the two treatments and the maximum decrease occurring after 60 min (Costello et al., 2012b). Similar results were obtained on changes in T_sk_ at the patellar region; a greater drop was observed with WBC immediately after the procedure, while 10–60 min after the treatment a lower temperature was reached with CWI (Costello et al., 2014). Interestingly, the authors had set the question whether or not either WBC or CWI were capable of achieving the T_sk_ (<13°C) believed to be required for analgesic purposes (Bleakley and Hopkins, 2010), yet they concluded that this temperature was reached by neither of the two procedures (Costello et al., 2014). Zalewski et al. confirmed that the maximum drop in core temperature occurred 50–60 min post-WBC (Zalewski et al., 2014).

In a systematic review, comparing 10 controlled trials, considering either a 10 min-long ice pack application, 5 min CWI, or 2.5–3 min WBC (−110° to −195°C), the authors illustrated that the largest reduction in T_sk_ was obtained by the ice pack application due to the higher heat transfer constant (*k* = 2.18) compared to water (*k* = 0.58) and air (*k* = 0.024). The obtained results confirmed negligible intramuscular temperature variation regardless of the cooling modality as well as importance of adiposity in determining cooling efficiency (*k* = 0.23 vs. *k* = 0.46 of muscles; Bleakley et al., 2014).

In summary, the following reports have been made about the WBC treatment:

- WBC is a medical practice that must be performed in specialized facilities under supervision of a well-trained personnel.
- WBC has contraindications that must be considered before prescription.
- Cooling efficiency and, possibly, treatment effectiveness can be influenced by body composition.
- Due to differences in body composition, cooling efficiency is potentially greater in females than in males.
- WBC effectiveness in lowering T_sk_ exceeds that of CWI; muscle and core temperatures seem to decrease in a similar way in response to both treatments.
- The maximum decrease in core temperature has been noted 50–60 min post-WBC.

## Hematology

The study of hematological response to WBC allows to define a wide range of effects covering modification of oxygen supply potential, inflammatory response, and coagulation function.

### Erythrocytes and hemoglobin

We studied hematological parameters, including iron metabolism ones, in 27 athletes belonging to National Italian Rugby Team, during a summer camp (Lombardi et al., 2013a). Two daily sessions of WBC (3 min, −140°C) were performed for seven consecutive days, one in the morning before the first training session, the second in the evening after the second training session. Athletes were strictly controlled for diet, especially the correct iron uptake. A typical plasma volume shift due to a prolonged training session of aerobic exercises was taken into account when interpreting the results. Among hematological parameters, erythrocytes (RBC), hematocrit (Ht), and hemoglobin (Hb) decreased noticeably; particularly, Hb decreased from 15.06 ± 0.84 to 14.70 ± 0.62 g/dL. Red cell distribution width (RDW) increased, indicating a rise of anisocytosis of RBC, although reticulocytes were stable, but the immature fraction of reticulocytes (IRF) was significantly decreased (Lombardi et al., 2013a). A decrease of hemoglobinization could be a specific feature of the WBC treatment. Indeed, a similar decrease of Hb (about 0.3 g/dL) and IRF had been previously reported in rugby players, however, in that case, RBC and Ht had not been affected (Banfi et al., 2008). This difference could be attributable to a milder WBC protocol, with only five WBC (one per day, at −110°C). The decrease in the levels of Hb as well as RBC and Ht, is transitory and it recovered during continuative treatments as demonstrated by Szygula and colleagues in a study performed on students of the Polish National Military Academy, who can be considered physically active subjects, continuously performing exercises and controlled for variables as diet and lifestyle (Szygula et al., 2014). Recruited cadets were divided into two groups of 15 subjects; one group was treated with WBC, the other did not receive the treatment. Hematological parameters were measured after 10, 20, and 30 sessions, which were performed daily in a cryochamber at −130°C, for 3 min. After 10 sessions, Hb decreased from a mean of 15.1 ± 0.74–14.4 ± 0.94 g/dL and remained at this concentration after 20 sessions (14.5 ± 0.71 g/dL). It then rose to 15.1 ± 1.1 g/dL after 30 sessions. Similar changes were observed for Ht and RBC. The decrease of Hb, RBC and Ht lasted through 20 sessions of the WBC treatment; then the bone marrow reacted by releasing new RBCs (Szygula et al., 2014). A decrease in Hb and RBC was already described in elite Polish field hockey players after 18 sessions of WBC (Straburzyńska-Lupa et al., 2007). Hb also showed a decreasing though not statistically significant trend, dropping from 15.0 ± 1.0 to 14.4 ± 0.8 g/dL, in nine collegiate physically active subjects, who had completed 30 min step up/down exercise, aimed at inducing delayed-onset muscle soreness (DOMS), and had been treated with two daily WBC sessions for 5 consecutive days. In opposite, the control group, which had undergone the same DOMS-inducing training without the WBC or any other recovery treatment, experienced stable levels of Hb (Ziemann et al., 2014). Nevertheless, some data revealed that Hb and RBC were stable in 12 professional tennis players, following 10 sessions of WBC applied twice a day, at –120°C for 3 min, over 5 days, during a controlled training camp (Ziemann et al., 2012) as well as in 16 kayakers treated twice a day for the first 10 days of a 19 day physical training cycle (Sutkowy et al., 2014). It is thus, possible that shifts in Hb and RBC induced by WBC are dependent on the discipline and baseline hematological profile. This issue, however, still has not been investigated. Mean curpuscular volume (MCV) grew following the WBC treatment applied in rugby players and in field hockey players (Straburzyńska-Lupa et al., 2007; Lombardi et al., 2013a); in the latter group values of MCV, mean curpuscular hemoglobin (MCH), and of mean curpuscular hemoglobin concentration (MCHC) remained elevated up to a week after the end of the treatment (Straburzyńska-Lupa et al., 2007).

A slight dehemoglobinazion has two direct consequences. Firstly, since the OFF-score, a parameter used to calculate the probability of blood doping in athletes, depends on Hb concentration and Ret count (Sottas et al., 2010; Robinson et al., 2011; which remained stable), WBC may reduce the result of this score and, thus, cannot be considered a performance enhancing practice. On the other hand, the use of WBC to mask illicit practices is unjustified because the potential decrease in Hb is too small and the change itself is short-lasting and/or temporary (Lombardi et al., 2013a). Secondly, the decrease in Hb and RBC should be considered when the timeline of recovery strategies, within a competitive season, is drawn.

### Iron metabolism

Martial status was not modified after the treatment in 27 rugby players submitted to two daily WBC sessions for 7 consecutive days (Lombardi et al., 2013a). Only soluble transferring receptor (sTfR) increased significantly, but not pathologically, possibly demonstrating initial high functional iron demand (Lombardi et al., 2013b). Similar results were obtained in a more recent paper by Dulian and colleagues. Regardless of the fitness level, in a cohort of obese subjects (BMI > 30 kg/m^2^), serum iron and ferritin remained unchanged after the 1st and 10th WBC session. Only hepcidin, a hepatocyte-derive peptide hormone mediating iron depletion in inflammation (Lombardi et al., 2013b), decreased moderately (Dulian et al., 2015).

### Hemolysis

WBC enhances hemolysis, which could explain the Hb decrease during initial phase of the treatment. A decrease of haptoglobin, scavenger protein for free Hb released from broken RBC was described in the above-mentioned paper by Szygula and co-workers, after 10 and 20 WBC sessions, but a recovery appeared after 30 sessions, following the changes in Hb and RBC. Contemporarily, bilirubin increased, reflecting Hb catabolism. Hemolysis stimulated release of erythropoietin (EPO), which increased by 4.5% compared to baseline after 10 sessions, and further by 10.8 and 10.1% after 20 and 30 sessions, respectively, possibly supporting the recovery of RBC number after the initial decrease. Even in the case of EPO, the shifts in concentrations remained within physiological ranges (Szygula et al., 2014).

### Leukocytes

Levels of leukocytes did not show any changes after 14 sessions of WBC (twice a day, over 7 days) in the group of 27 rugby players, belonging to National Italian Rugby Team, studied during a summer camp (Lombardi et al., 2013a). The same was found for the group of 16 kayakers treated twice a day for the first 10 days of a 19 day physical training cycle (Sutkowy et al., 2014).

At the same time, leukocytes increased in the students of the Polish Military Academy after 10 and 20 sessions, but returned to baseline values after 30 sessions. The increase trend covered both granulocytes and lymphocytes (Szygula et al., 2014). Similar increase was also reported in tennis players, but not for subcategories of granulocytes and lymphocytes (Ziemann et al., 2012). Despite the increase, leukocytes always remained within the physiological range. Mobilization of leukocytes from the bone marrow and organs of residence has been hypothesized as a possible cause of these increases although an explanation of this phenomenon is still lacking.

In endurance trained runners, a simulated 45 min trail run, designed specifically to trigger exercise-induced muscle damage (EIMD), followed by four sessions of WBC applied once a day, resulted in an increase in neutrophil count of 114% compared to baseline, with the maximum peak recorded 1 h after the exercise. The correspondent increase in neutrophils, following passive recovery, accounted for 101% shift against baseline. The authors hypothesized that the increase of circulating neutrophils stimulated angiogenesis (via vascular endothelial growth factor—VEGF expression) and the consequent improved perfusion was associated with a reduced delayed onset of muscle soreness (DOMS) and, hence, an improved recovery (Pournot et al., 2011).

### Platelets

Platelets did not shift in response to WBC sessions applied in groups of rugby and tennis players (Lombardi et al., 2013a; Ziemann et al., 2014) nor students of the Polish Military Academy (Szygula et al., 2014).

In summary, the following reports have been made about the WBC treatment:

- WBC causes a decrease in Hb, Ht, and RBC after 5, 10, and 20 sessions. A recovery of hemoglobinization is reached after 30 sessions. Ret counts remains unaffected by WBC.
- The effect of WBC on RBC and Hb can be influenced by the type and intensity of physical training since in some groups of athletes these changes did not occur.
- Hemolysis may be the cause behind the drop in RBC, Hb, and Ht following the WBC treatment of 10–20 sessions.
- EPO is induced in the course of WBC with the aim to recover to baseline levels of RBC and Hb.
- WBC should not have a boosting effect on bone marrow and is not influencing athletes' hematological parameters usually controlled to test for illicit bone marrow stimulation.
- The level of leukocytes either does not change or only slightly increases in response to WBC. Cryotherapy possibly mobilizes leukocytes, especially neutrophils, with a positive effect on DOMS.
- Platelets are not affected by WBC.

## Lipids concentrations and energy metabolism

Lipids are the main source of energy as well as the main thermogenic substrate. It is thus, possible that an intense cold stimulus of WBC affects lipid metabolism.

Sixty-nine physically active male subjects (10 professional rugby players and 39 healthy individuals including policemen and soldiers) were divided into three groups based on the number of WBC sessions (−130°C, 3 min): (i) 5, (ii) 10, and (iii) 20. Five sessions of WBC did not modify the lipid profile. Ten sessions induced a 34% decrease in triglycerides and much more after 20 sessions (from 108 ± 50 mg/dL, before the start of the treatment, to 69.4 ± 27.2 mg/dL, after 20 sessions). After 20 sessions, high density lipoprotein (HDL) cholesterol significantly increased (from 53.2 ± 16.5 to 63.1 ± 27.4 mg/dL), whilst low density lipoprotein (LDL) cholesterol decreased noticeably (from 97.7 ± 48.3 to 72.8 ± 52.0 mg/dL; Lubkowska et al., 2010a). Concentrations of total cholesterol and LDL also decreased in physically active males, who underwent a 30 min step up/down exercise supplemented with WBC applied twice a day over 5 consecutive days; compared to the control group, total cholesterol and LDL dropped by 43 and 52%, respectively (Ziemann et al., 2014).

Adipose tissues, both white (WAT) and brown (BAT), become activated during cold exposure. BAT is particularly consumed during exposure to cold, contributing to energy metabolism. However, 6 months of moderate aerobic activity combined with WBC did not change body mass, fat, or lean body mass percentages. Circulating adiponectin, leptin, and resistin concentrations were also not modified while, again, LDL and triglycerides decreased and HDL increased. The experiment was performed on 45 overweight and obese men, asked to complete a test on a bicycle ergometer, beginning with a workload of 1 W/Kg of fat-free mass, then increased by 0.5 W/Kg half until exhaustion. Participants exercised three times a week under researchers' supervision; once a week they completed a 45 min-long walking session and, twice a week, attended a 45 min gym session. After 1 month from the start of the fitness program and 1 month before its end, 20 daily WBC treatments at −130°C for 3 min were done (Lubkowska et al., 2015).

Ten WBC sessions (−120°C, 3 min), applied twice day over 5 days, in 12 professional tennis players, during a controlled training camp, affected neither the resting metabolic rate nor the percentage of fat used as a metabolic substrate; this was also the case with healthy, physically active men treated with two daily WBC sessions, for 5 consecutive days between step up/down 30 min-long exercises (Ziemann et al., 2012, 2014).

Recently, a differential effectiveness of WBC has been implied to depend on the starting fitness status of the subject. Middle-aged obese men (BMI > 30 kg/m^2^) were grouped based on their fitness level (low and high cardiorespiratory fitness level) and exposed to 10 consecutive sessions of WBC over 2 weeks. Irisin, a myokine induced by exercise stimulating WAT browning, linked to enhanced thermogenic capacity (Boström et al., 2012; Lombardi et al., 2016), increased slightly in both groups over the course of 24 h after the first session of WBC. More interestingly, irisin plasma concentrations increased by 20% in subjects with a low fitness level, but decreased slightly in subjects with a high fitness level (Dulian et al., 2015). The observed increase in irisin corresponds with data of Lee and co-workers, who investigated changes of myokine—irisin and adipokine—fibroblast growth factor 21 (FGF21) in response to cold water immersion. In contrast to trends in irisin, they observed a greater reduction in FGF21, interpreted as a pronounced effect of the non-shivering thermogenesis (Lee et al., 2014). There are no data about changes in FGF21 in response to WBC.

In summary, the following reports have been made about the WBC treatment:

- WBC has a dose-dependent improving effect on the lipid profile.
- WBC does not affect the rest metabolic rate and energy expenditure during exercise.
- WBC has a stimulatory effect on irisin expression, which should act at the adipose tissue level by enhancing thermogenesis.

## Bone metabolism and skeletal health

Bone health is essential not only in whole-body health, but also in determining and sustaining athletes' performance (Lombardi et al., 2016; Sansoni et al., 2017). Due to high energy expenses associated with high-level physical activity (Lombardi et al., 2012), the risk of osteopenia and stress fractures (the inability of the skeleton to modify its own microarchitecture depending on applied loads) is always present. Based on these findings, the availability of medical tools favoring recovery of the bone tissue is highly desirable (Banfi et al., 2010a; Lombardi et al., 2011).

Ten professional rugby players, belonging to Italian National Team, submitted to single daily sessions of WBC for 5 consecutive days (−110°C, 2 min), were compared to 10 players, who completed the same training protocol without WBC (Galliera et al., 2012). Bone metabolism was studied through biochemical parameters. The soluble ligand of the receptor activator of nuclear factor κB (RANKL) and its decoy receptor osteoprotegerin (OPG) constitute a fundamental cytokine system connecting the immune system and bone metabolism in order to link pro- and anti-inflammatory balance to calcium stores. RANKL is released from osteoblasts and lymphocytes and activates osteoclasts, inducing bone resorption. Osteoclasts express RANK, specific receptor of RANKL, which induces an intracellular signal to reabsorb bone. The effect of RANKL on RANK is blocked by OPG (Banfi et al., 2010a). WBC did not affect plasma RANK and RANKL concentrations in athletes, but increased OPG, thus, causing the OPG/RANKL ratio, an index of resorption-to-formation balance, to grow as well. The increased osteogenic potential may have a role in the post-fracture recovery, but also in prevention of more insidious stress fractures (Galliera et al., 2012).

In summary, the following reports have been made about the WBC treatment:

- WBC could counteract the inflammation-induced bone resorption.

## Inflammatory markers

The anti-inflammatory and analgesic effects of cryotherapy are the most searched for by athletes and patients. The effectiveness of the treatment in this specific ambit has been demonstrated by a number of studies although the available data is contrasting and still debated. Indeed, along with studies reporting direct effect of WBC on inflammatory markers, there are several reports that did not link any changes with the treatment at all. Nevertheless, almost all of these studies agree on general benefits induced by the treatment including improved pain, mood, and quality of life (QoL). A prototypic example was found in patients affected by fibromyalgia, an auto-inflammatory disease of unknown pathogenesis and highly variable clinical presentation, always characterized by chronic systemic inflammation, generalized pain, and severe fatigue, which invariably and considerably deteriorates the QoL; regardless of the conventional analgesic therapy, compared to subjects not treated with WBC (*n* = 50), patients receiving the WBC treatment (*n* = 50) exhibited large clinical improvements in all the investigated parameters depicting the QoL and the ability to perform daily activities [pain by visual analog scale (VAS), global health status (GH), fatigue severity scale (FSS), and short form (SH)-36; Bettoni et al., 2013].

Tumor necrosis factor (TNFα), interleukin 6 (IL-6), and IL-10 did not change in 11 runners, who underwent a 48-min pro-EIMD training protocol and were treated four times with either WBC at −110°C or passive recovery. On the contrary, the increase in the pro-inflammatory marker IL-1β 24 h post treatment and C-reactive protein (CRP) 96 h post-WBC, were strongly attenuated compared to passive recovery. In parallel, WBC had a greater inducing effect on the anti-inflammatory IL-1 receptor antagonist (IL-1ra) 1 h post-treatment. The authors concluded that a unique session of WBC (3 min at −110°C), performed immediately after exercise, enhanced muscular recovery by restricting the inflammatory process (Pournot et al., 2011). Five days of WBC applied twice a day (−120°C, 3 min), combined with moderate-intensity training, in six professional tennis players, during a controlled training camp, decreased TNFα serum concentrations by 60% as opposed to 35% decrease observed in the untreated group. IL-6, instead, increased slightly, but significantly due to WBC (Ziemann et al., 2012). The pro-inflammatory role of IL-6 has been recently revised (Peake et al., 2015). Indeed, it has a dual effect depending on the production site and its basal concentration. Chronically high, even if moderately, IL-6, characterizing chronic inflammatory conditions (e.g., obesity, sedentariness) stimulating the hepatic synthesis of this cytokine, cause pro-inflammatory, and potentially deleterious effect. On the contrary, even very high, pulsatile spikes of IL-6, originating from low basal concentrations, derived from contracting muscles, act as a powerful anti-inflammatory mediator (Lombardi et al., 2016). In 18 professional male volleyball players, a session of submaximal exercise increased IL-1β and IL-6 by 60%. The WBC treatment (−130°C, 3 min), however, performed before the exercise, prevented these increases. This study, thus, highlighted a possible preventive effect of WBC on exercise-induced inflammation (Mila-Kierzenkowska et al., 2013). However, in rugby players, a single session of WBC (−130°C) did not affect IL-6 values, regardless of the treatment‘s duration (1, 2, or 3 min; Selfe et al., 2014). The protective effect of WBC was also demonstrated in 18 physically active college-aged men, who underwent eccentric workout inducing DOMS. A single session of WBC (−110°C, 3 min) performed after the exercise had the same effect of a passive recovery (increased IL-6, IL-1β, and IL-10). After 5 days, the repeated performed exercise induced the similar biochemical modifications in the untreated group, while in the WBC-treated group (5 days, twice daily) IL-6 and IL-1β were unchanged compared to baseline with IL-10 strongly elevated (Ziemann et al., 2014).

In healthy young men, the extent of IL-6 increase, 30 min and 24 h after a single session of WBC, was much greater than the corresponding increases observed after 10 days of the treatment (Lubkowska et al., 2010b). Interestingly, the same authors also demonstrated that in 45 healthy men, a different number of sessions had a different effect on particular inflammatory parameters. Five sessions of WBC increased IL-10 by 30%, but this change was dissipated 2 weeks after the end of the treatment. After 10 sessions IL-1α decreased by 17%, while IL-6 and IL-10 increased by 10% and 14% respectively, yet even in this case, these modifications were lost after 2 weeks. After 20 sessions of WBC the cytokine balance was confirmed just like after 10 sessions, but in this case, the decrease in IL-1α was sustained 2 weeks after the end of the treatment. IL-1β, TNFα, and IL-12 remained unchanged over the observation period. Consequently, the authors suggested to use 20 sessions in order to induce adaptation (Lubkowska et al., 2011). In professional rugby players, a slight, yet not significant decrease in ferritin and transferrin concentrations was observed that, together with an increase in the sTfR concentration, further supported the anti-inflammatory effect of WBC. Indeed, these parameters generally have a contrary behavior, when a chronic inflammatory process is ongoing (Lombardi et al., 2013b). Interestingly, as for hematological parameters and martial status, a shift in the cytokine profile depends upon fitness capacity. Pro-inflammatory cytokines (IL-6, TNFα) and adipokines (resistin, visfatin) were higher in obese LCF subjects compared to the obese HCF ones. IL-10 increased equally in both groups, with adiponectin and leptin unaffected (Ziemann et al., 2013). Even the drop in CRP, observed regardless of the number of sessions (either 1 or 10), was much greater in obese LFL than in obese HFL (Dulian et al., 2015). The anti-inflammatory effect of WBC is in line with previous reports (Banfi et al., 2009b).

Table 1 presents the findings about the effects of WBC on circulating levels of cytokines and inflammatory markers.

**Table 1:** **Effects of WBC on circulating levels of cytokines and inflammatory markers**.

**Subjects**	**WBC mode**	**WBC sessions**	**Effects**	**References**
**IL-6**				
15 healthy men (age: 22.0 ± 0.8 years, BMI 23.2 ± 1.4 kg/m^2^)	3 min, −130°C	5 sessions	Increased at the end of the treatment compared to baseline	Lubkowska et al., 2011
		1 per day (morning)		
		5 consecutive days	No difference 2 weeks after the end of treatment compared to baseline	
15 healthy men (age: 22.0 ± 0.8 years, BMI 23.2 ± 1.4 kg/m^2^)	3 min, −130°C	10 sessions	Increased at the end of the treatment compared to baseline	Lubkowska et al., 2011
		1 per day (morning)		
		2 weeks^*^	No difference 2 weeks after the end of treatment compared to baseline	
15 healthy men (age: 22.0 ± 0.8 years, BMI 23.2 ± 1.4 kg/m^2^)	3 min, −130°C	20 sessions	Increased at the end of the treatment compared to baseline	Lubkowska et al., 2011
		1 per day (morning)		
		4 weeks^*^	No difference 2 weeks after the end of treatment compared to baseline	
12 tennis players (6 treated, age: 23.0 ± 3.0 years, BMI 23.2 ± 1.8; 6 Kg/m^2^ untreated, 20.0 ± 2.0 years, BMI 24.4 ± 1.9 kg/m^2^)	3 min, −120°C	10 sessions	Increased at the end of the treatment compared to baseline	Ziemann et al., 2012
		2 per day		
		5 consecutive days	Significant difference vs. controls	
12 men, obese (age: 38.4 ± 8.2 years, BMI > 30 kg/m^2^, divided in HFL group and in LFL group, visceral fat area >100 cm^2^)	3 min, −110°C	10 sessions (morning after light breakfast)	No effect on HFL group	Dulian et al., 2015
			No effect on LFL group	
			No difference between the groups	
11 runners (age: 31.8 ± 2.0 years, simulated trail with WBC treatment or passive recovery, blood drawings before and after the trail, and after 1, 24, 48, 72, and 96 h during recovery)	3 min, −110°C	4 sessions	No effect	Pournot et al., 2011
14 obese men (age: 40.0 ± 4.0 years, divided in HFL group and in LFL group)	3 min, −110°C	10 sessions (morning after light breakfast)	Decreased in LFL group compared to baseline	Ziemann et al., 2013
18 volleyball players (age: 28.3 ± 4.0 years)	2 min, −130°C	1 session	Decreased at the end of the treatment compared to baseline	Mila-Kierzenkowska et al., 2013
14 rugby players (Mean age: 24 years)	1, 2, 3 min, −135°C	1 session	No effect	Selfe et al., 2014
18 men (9 treated, age: 21.7 ± 0.9 years; 9 untreated, age: 22.0 ± 2.0 years)	3 min, −110°C	10 sessions	No effect	Ziemann et al., 2014
		2 per day		
		5 consecutive days		
**IL-10**				
15 healthy men (age: 22.0 ± 0.8 years, BMI 23.2 ± 1.4 kg/m^2^)	3 min, −130°C	5 sessions	Increased at the end of the treatment compared to baseline	Lubkowska et al., 2011
		1 per day (morning)		
		5 consecutive days	Increase 2 weeks after the end of treatment compared to baseline	
15 healthy men (age: 22.0 ± 0.8 years, BMI 23.2 ± 1.4 kg/m^2^)	3 min, −130°C	10 sessions	Increased at the end of the treatment compared to baseline	Lubkowska et al., 2011
		1 per day (morning)		
		2 weeks^*^	No difference 2 weeks after the end of treatment compared to baseline	
15 healthy men (age: 22.0 ± 0.8 years, BMI 23.2 ± 1.4 kg/m^2^)	3 min, −130°C	20 sessions	Increased at the end of the treatment compared to baseline	Lubkowska et al., 2011
		1 per day (morning)		
		4 weeks^*^	Increased 2 weeks after the end of treatment compared to baseline	
11 runners (age: 31.8 ± 2.0 years, simulated trail with WBC treatment or passive recovery, blood drawings before and after the trail, and after 1, 24, 48, 72, and 96 h during recovery)	3 min, −130°C	4 sessions	No effect	Pournot et al., 2011
14 obese men (age: 40.0 ± 4.0 years, divided in HFL group and in LFL group)	3 min, −110°C	10 sessions (morning after light breakfast)	Increased in both groups compared to baseline	Ziemann et al., 2013
18 men (9 treated, age: 21.7 ± 0.9 years; 9 untreated, age: 22.0 ± 2.0 years)	3 min, −110°C	10 sessions	Increased compared to baseline	Ziemann et al., 2014
		2 per day		
		5 consecutive days		
**IL-12**				
15 healthy men (age: 22.0 ± 0.8 years, BMI 23.2 ± 1.4 kg/m^2^)	3 min, −130°C	5 sessions	No effect at the end of the treatment compared to baseline	Lubkowska et al., 2011
		1 per day (morning)		
		5 consecutive days	No difference 2 weeks after the end of treatment compared to baseline	
15 healthy men (age: 22.0 ± 0.8 years, BMI 23.2 ± 1.4 kg/m^2^)	3 min, −130°C	10 sessions	No effect at the end of the treatment compared to baseline	Lubkowska et al., 2011
		1 per day (morning)		
		2 weeks^*^	No difference 2 weeks after the end of treatment compared to baseline	
15 healthy men (age: 22.0 ± 0.8 years, BMI 23.2 ± 1.4 kg/m^2^)	3 min, −130°C	20 sessions	No effect at the end of the treatment compared to baseline	Lubkowska et al., 2011
		1 per day (morning)		
		4 weeks^*^	No difference 2 weeks after the end of treatment compared to baseline	
**IL-1**α				
15 healthy men (age: 22.0 ± 0.8 years, BMI 23.2 ± 1.4 kg/m^2^)	3 min, −130°C	5 sessions	Decreased at the end of the treatment compared to baseline	Lubkowska et al., 2011
		1 per day (morning)		
		5 consecutive days	No difference 2 weeks after the end of treatment compared to baseline	
15 healthy men (age: 22.0 ± 0.8 years, BMI 23.2 ± 1.4 kg/m^2^)	3 min, −130°C	10 sessions	Decreased at the end of the treatment compared to baseline	Lubkowska et al., 2011
		1 per day (morning)		
		2 weeks^*^	No difference 2 weeks after the end of treatment compared to baseline	
15 healthy men (age: 22.0 ± 0.8 years, BMI 23.2 ± 1.4 kg/m^2^)	3 min, −130°C	20 sessions	Decreased at the end of the treatment compared to baseline	Lubkowska et al., 2011
		1 per day (morning)		
		4 weeks^*^	No difference 2 weeks after the end of treatment compared to baseline	
**IL-1**β				
15 healthy men (age: 22.0 ± 0.8 years, BMI 23.2 ± 1.4 kg/m^2^)	3 min, −130°C	5 sessions	No effect at the end of the treatment compared to baseline	Lubkowska et al., 2011
		1 per day (morning)		
		5 consecutive days	No difference 2 weeks after the end of treatment compared to baseline	
15 healthy men (age: 22.0 ± 0.8 years, BMI 23.2 ± 1.4 kg/m^2^)	3 min, −130°C	10 sessions	No effect at the end of the treatment compared to baseline	Lubkowska et al., 2011
		1 per day (morning)		
		2 weeks^*^	No difference 2 weeks after the end of treatment compared to baseline	
15 healthy men (age: 22.0 ± 0.8 years, BMI 23.2 ± 1.4 kg/m^2^)	3 min, −130°C	20 sessions	No effect at the end of the treatment compared to baseline	Lubkowska et al., 2011
		1 per day (morning)		
		4 weeks^*^	No difference 2 weeks after the end of treatment compared to baseline	
11 runners (age: 31.8 ± 2.0 years, simulated trail with WBC treatment or passive recovery, blood drawings before and after the trail, and after 1, 24, 48, 72, and 96 h during recovery)	3 min, −110°C	4 sessions	Significantly lower in WBC vs. passive recovery after 1 and 24 h	Pournot et al., 2011
18 volleyball players (age: 28.3 ± 4.0 years)	2 min, −130°C	1 session	Decreased compared to baseline	Mila-Kierzenkowska et al., 2013
18 men (9 treated, age: 21.7 ± 0.9 years; 9 untreated, age: 22.0 ± 2.0 years)	3 min, −110°C	10 sessions	Decreased in treated subjects compared to untreated subjects	Ziemann et al., 2014
		2 per day		
		5 consecutive days		
**IL-1ra**				
11 runners (age: 31.8 ± 2.0 years, simulated trail with WBC treatment or passive recovery, blood drawings before and after the trail, and after 1, 24, 48, 72, and 96 h during recovery)	3 min, −110°C	4 sessions	Decreased	Pournot et al., 2011
		Significantly lower in WBC vs. passive recovery after 1 h		
**URIC ACID**				
46 subjects (24 M, 22 F; age: 37.5 ± 3.1 years; BMI: 26.9 ± 4.2 kg/m^2^)	3 min, −130°C	10 sessions	Increased in both males and females compared to baseline	Miller et al., 2012
		1 per day		
		2 weeks^*^		
30 men (age: 27.8 ± 6.1 years; BMI: 22.1–33.2 kg/m^2^)	3 min, −130°C	20 sessions	No effect after 1 session	Lubkowska et al., 2012
		1 per day (morning)		
		20 consecutive days	Increased after 10 and 20 sessions	
**IL-3**				
45 men from Military Academy (age: 23.5 ± 0.8 years; BMI: 25.0 ± 2.1 kg/m^2^); 30 treated vs. 15 untreated	3 min, −130°C	30 sessions	Decreased after 10, 20, and 30 sessions	Szygula et al., 2014
		1 per day (morning)		
		5 consecutive days	Significant differences between groups at each time-point	
**TNF**α				
15 healthy men (age: 22.0 ± 0.8 years, BMI 23.2 ± 1.4 kg/m^2^)	3 min, −130°C	5 sessions	No effect at the end of the treatment compared to baseline	Lubkowska et al., 2011
		1 per day (morning)		
		5 consecutive days	No difference 2 weeks after the end of treatment compared to baseline	
15 healthy men (age: 22.0 ± 0.8 years, BMI 23.2 ± 1.4 kg/m^2^)	3 min, −130°C	10 sessions	No effect at the end of the treatment compared to baseline	Lubkowska et al., 2011
		1 per day (morning)		
		2 weeks^*^	No difference 2 weeks after the end of treatment compared to baseline	
15 healthy men (age: 22.0 ± 0.8 years, BMI 23.2 ± 1.4 kg/m^2^)	3 min, −130°C	20 sessions	No effect at the end of the treatment compared to baseline	Lubkowska et al., 2011
		1 per day (morning)		
		2 weeks^*^	No difference 2 weeks after the end of treatment compared to baseline	
12 tennis players (6 treated, age: 23.0 ± 3.0 years, BMI 23.2 ± 1.8 Kg/m^2^; 6 untreated, 20.0 ± 2.0 years, BMI 24.4 ± 1.9 kg/m^2^)	3 min, −120°C	10 sessions	Decreased compared to baseline	Ziemann et al., 2012
		2 per day		
		5 consecutive days	Significant difference vs. controls	
11 runners (age: 31.8 ± 2.0 years, simulated trail with WBC treatment or passive recovery, blood drawings before and after the trail, and after 1, 24, 48, 72, and 96 h during recovery)	3 min, −110°C	4 sessions	No effect	Pournot et al., 2011
14 obese men (age: 40.0 ± 4.0 years, divided in HFL and LFL)	3 min, −110°C	10 sessions (morning after light breakfast)	Decreased in LFL group compared to baseline	Ziemann et al., 2013
18 volleyball players (age: 28.3 ± 4.0 years)	2 min, −130°C	1 session	No effect	Mila-Kierzenkowska et al., 2013
**hsCRP**				
12 men, obese (age: 38.4 ± 8.2 years, BMI > 30 kg/m^2^, divided in HFL and LFL, visceral fat area > 100 cm^2^)	3 min, −110°C	10 sessions, in the morning, preceded by light breakfast	Decreased 30 min and 24 h after 1 session in both groups	Dulian et al., 2015
		Decreased 30 min and 24 h after 10 sessions in both groups		
**RESISTIN**				
14 obese men (Age: 40.0 ± 4.0 years, divided in HFL and LFL)	3 min, −110°C	10 sessions (morning after light breakfast)	Increased in HFL group compared to baseline	Ziemann et al., 2013
		Decreased in LFL group compared to baseline		
**VISFATIN**				
14 obese men (age: 40.0 ± 4.0 years, divided in HFL and LFL)	3 min, −110°C	10 sessions (morning after light breakfast)	Increased in HFL group compared to baseline	Ziemann et al., 2013
		Decreased in LFL group compared to baseline		

* *Excluding Saturday and Sunday; HFL, high fitness level; LFL, low fitness level*.

It is worth noting that cooling therapies are often applied interchangeably in athletes' recovery treatment. Reducing inflammation may have another interesting aspect when achieved in athletes. The latest paper published by Roberts and co-workers revealed that a cooling therapy (cold water immersion-CWI: 10 min using a circulatory cooling unit, temperature at 10.1 ± 0.3°C) used after resistance training as a recovery strategy attenuates both acute changes in satellite cell numbers and kinase activity that regulates muscle hypertrophy, which may translate into smaller, but longer-term training gains in muscle strength and hypertrophy. Consequently, reduced inflammation in response to WBC may have a negative effect on muscle hypertrophy (Roberts et al., 2015).

In summary, the following reports have been made about the WBC treatment:

- WBC induces anti-inflammatory effects.
- Findings about the effect of WBC on IL-6 are not always concordant, probably due to differences in exercise protocols applied. In general terms, a single session of WBC increases IL-6 concentration, while multiple sessions recover it to baseline. In this sense, WBC seems to mimic exercise-induced impacts.
- More consistently, WBC stimulates the anti-inflammatory response (reduced IL-1β and increased IL-10, IL-1Ra).
- Fitness capacity affects the inflammatory response to WBC in non-athletes.
- Further, investigations should focus on establishing, whether reduced inflammation has a beneficial effect on athletes' performance if they combine training and cold therapy.

## Endocrine function and hormone profile

Monitoring hormones is essential to assess health status especially in athletes, who experience chronic intense workloads associated with psychophysically stressful situations. WBC is used to relieve stress conditions owing to the activation of neuroendocrine and metabolic axes regulating thermal homeostasis. However, only a few of reports published so far had considered this aspect.

Salivary steroid hormones were monitored in 25 professional top level rugby players, during a summer training camp. The athletes were submitted to the WBC treatment (−140°C, 3 min) twice a day for 7 consecutive days, the first before the morning training session, the second after the evening workout. Saliva was collected before the start of the camp, after the evening WBC session on the first day, and after the last WBC session on the last day. Compared to baseline, at the end of the first day (2 WBC sessions completed) cortisol and dehydroepiandrosterone (DHEA) decreased. After 7 days, cortisol, DHEA, and estradiol decreased, yet testosterone increased. Importantly, in the majority of subjects variations exceeded the critical difference (CD). CD is the minimal value, calculated between two consecutive measurements of the same parameter, using the same method, on the same individual and including analytical and biological variabilities, which when exceeded, testifies that an external factor is really modifying the investigated parameter. Notably, the testosterone-to-cortisol ratio, widely accepted as an index of the potential athletic performance status, increased as a result of the observed changes (Grasso et al., 2014).

Cortisol increased in six professional tennis players treated with WBC twice a day for 5 days (−120°C, 3 min) after moderate-intensity training, during a controlled training camp (Ziemann et al., 2012), while testosterone remained stable. Cortisol and testosterone were both stable in 16 kayakers from Polish National Team, who combined exercise with two daily WBC sessions during the first 10 days of a training cycle in preparation for the World Championships (Sutkowy et al., 2014). Six elite rowers were subject to a 6 day training cycle combined with training sessions scheduled twice daily, each preceded by WBC (−125/−150°C, 3 min). During a control training session without WBC, subjects experienced an increase in cortisol on the 3rd day of training; WBC delayed and reduced this increase on the 6th day (Wozniak et al., 2013). After a normal training week, 10 elite synchronized swimmers performed two 2 week sessions of an intensified training, randomly combined either with or without daily WBC. The two sessions were separated by a 9 day light training period. Although salivary cortisol was unchanged, WBC mitigated signs of functional overreaching observed during the control period such as reduced sleep quantity, increased fatigue and impaired exercise capacity (Schaal et al., 2015).

A randomized, counterbalanced and crossover study on 14 habituated English Premier League academy soccer players, a single WBC session (−135°C, 2 min), performed within 20 min after a repeated sprint exercise (15 × 30 m), increased salivary testosterone up to 24 h post-exercise. Still, the treatment had no effect on cortisol, blood lactate, and CK (Russell et al., 2017).

In summary, the following reports have been made about the WBC treatment:

- WBC affects the hormonal asset, decreasing hormones typically associated with psychophysical stress, such as cortisol, and increasing testosterone, a typical anabolic hormone.
- Cortisol modifications during WBC treatment cycles are not consistent in the current literature, possibly owing to different stressors applied.

## Redox balance

Oxidative stress is the main factor affecting athletes' performance. Muscle activity generates oxidants released in the intercellular space following membrane leakage or breaking. Oxidants activate chain reactions, amplifying production of reactive oxygen species (ROS), which damage membranes, cellular structures and DNA. Inflammation activates innate immunity, which produces ROS and, through a vicious cycle, ROS-sustained inflammation (Slattery et al., 2015). WBC has been advocated to possibly enhance antioxidant capacities and, thus, counteract the exercise-induced ROS production.

The study on WBC effects on an oxidant-antioxidant balance was performed in 16 kayakers belonging to Polish National Team submitted to a 19 day physical training cycle. During the first 10 days of the training cycle, the kayakers combined exercise with two daily WBC sessions: the first in the morning before exercise, the second session in the afternoon after exercise. Day by day, the temperature was decreased from −120 to −145°C. Blood drawings were performed at the beginning of the training period as well as after 5, 11, and 19 days. Lipid peroxidation products malondialdehyde (MDA), conjugate dienes, and thiobarbituric acid reactive substances (TBARS) did not show any changes. Glutathione peroxidase (GPx) activity decreased after 5 days, yet later it increased again to baseline following WBC (11th day). On the last day of training, 9 days after the end of the WBC treatment, TBARS decreased compared to baseline and the level on day 5, whilst GPx increased compared to day 5. WBC may improve the efficiency of TBARS elimination that positively impacts strenuous exercise (Sutkowy et al., 2014).

Eighteen professional volleyball players performed the first 40 min submaximal exercise bout on an ergometer 2 weeks after the end of season, and the second bout 2 weeks after the first one. A single WBC treatment (−130°C) was conducted before the first exercise bout. The activity of RBC catalase after WBC + exercise was two times lower compared to that recorded after control exercise, without WBC, as was the activity of RBC superoxide dismutase (SOD; −8%; Mila-Kierzenkowska et al., 2013).

SOD and GPx activities were lower (by 44 and 42%, respectively) after 3 days of training combined with WBC than without the treatment in six elite rowers, subject to a 6 day training cycle. Lower levels of TBARS and conjugated dienes in erythrocytes were also found in the WBC-treated group (Wozniak et al., 2013).

In healthy subjects, 24 males and 22 females, after 10 daily WBC sessions, plasma uric acid, SOD, and total antioxidants all increased compared to both baseline and the control group not submitted to WBC. TBARS, although unchanged compared to baseline, were also higher than in the control group (Miller et al., 2012). During a 6 month long physical exercise program with two daily WBC exposures over 20 sessions, a significant decrease in SOD, catalase and glutathione reductase activities was observed (13, 8, and 70%, respectively). SOD activity increased after successive WBC sessions, while catalase activity progressively decreased (Lubkowska et al., 2015).

Although a general beneficial antioxidant effect already after a limited number of WBC sessions is described in these studies, a dose-dependency was observed in healthy men, with 20 sessions being the optimal number (Lubkowska et al., 2012).

In summary, the following reports have been made about the WBC treatment:

- WBC has a dose-dependent improving effect on the redox balance after exercise.
- WBC is able to decrease the activity of RBC enzymes probably as a reaction to a decrease in total oxidants and an increase in antioxidants.

## Muscle damage parameters, fatigue recovery, and pain

From athletes' and sports physicians' point of view the effect of WBC on muscle damage and recovery is the most important one. Indeed, muscle recovery has been the most claimed and, possibly, the most debated effect of WBC. In this specific field, the stimulatory effect of WBC is particularly claimed.

EIMD was studied in nine endurance runners, comparing three different recovery modalities: WBC, far-infrared (FIR), and passive. The runners performed three identical repetitions of a simulated trail run on a motorized treadmill. Recovery was evaluated 1, 24, and 48 h post-exercise. The eventual decrease in maximal isometric force, after three isometric voluntary contractions of knee extensor muscles, was used for judging muscle damage due to strenuous exercise. The best results were observed after WBC at 1, 24, and 48 h post-exercise. WBC-enhanced psychological recovery within days after the exercise including decreased perception of muscular tiredness and pain, already after the first session of WBC. At the same time the pain sensation was lowered by FIR only 48 h after the exercise and it did not change at all as a result of passive recovery. Well-being, evaluated through a standardized questionnaire for tiredness and pain, was improved after 24 h due to WBC and after 48 h due to FIR. Still, an increase in serum activity of creatine kinase (CK), typical of strenuous exercise, was not improved by three WBC sessions (Hausswirth et al., 2011).

A 40% decrease in CK activity was reported in rugby players after five consecutive daily sessions of WBC (Banfi et al., 2009b); it also decreased by 34% after 10 sessions in kayakers (Wozniak et al., 2007). The same decreasing trend of CK was also confirmed in 12 professional tennis players (CK dropped from 305.0 to 241.4 U/L in six treated subjects, while remained unchanged in the control group ones: 286.7 and 295.5 U/L), during a controlled training camp, during which players were treated with WBC twice a day for 5 days (−120°C, 3 min; Ziemann et al., 2012). The same was also noted in six elite rowers, subject to two 6 day training cycles with training sessions twice a day, all either preceded by WBC or not (Wozniak et al., 2013) as well as in physically active males treated with two daily WBC sessions for 5 consecutive days between step up/down 30 min exercises; in the latter case, the changes were also accompanied by a significantly improved pain perception (Ziemann et al., 2014). In professional rugby players, after a 7 day training camp with two daily WBC sessions, lactate dehydrogenase (LDH), and aspartate aminotransferase (AST) activities increased as a consequence of high workload, whilst CK decreased slightly, but significantly. Moreover, kidney function (estimated glomerular filtration rate, eGFR), which could be impaired following muscle damages, was unaffected by the treatment (Lombardi et al., 2014).

The WBC-induced attenuation in serum soluble intercellular adhesion molecule (sICAM)-1 immediately after EIMD may be responsible for reduced acute inflammatory response muscle damage (Ferreira-Junior et al., 2014). The level of sICAM-1 together with CK and LDH activities decreased in rugby players after 5 consecutive days of daily WBC sessions (Banfi et al., 2009b). After muscle damage is induced by exercise, leukocytes are mobilized to injured tissues by sICAM-1 intervention, where pro-inflammatory cytokines and ROS are released. WBC causes vasoconstriction and reduces the number of leukocytes reaching the muscles by inhibiting sICAM-1 (Ferreira-Junior et al., 2014). However, it was also hypothesized that cold could induce an extra-release of sICAM1 and, therefore, cause a decrease of muscle inflammation; the final net effect is the same (Dugué, 2015).

Beneficial effects of WBC on psychological recovery within days after exercise included a decreased perception of muscular tiredness and pain- an greater-improvement compared to the effect of other recovery modes, namely FIR and passive (Pournot et al., 2011). Daily WBC in 10 top level female synchronized swimmers (−110°C, 3 min), applied following training sessions, improved athletes' tolerance to training load by preserving sleep quantity during the period of intensive training before the Olympic Games (Schaal et al., 2015). The effect was evaluated after the athletes had been randomly assigned either WBC or non-WBC supported recovery. WBC use had a beneficial effect on sleep duration, limiting sleep latency possibly conditioned by post-exercise parasympathetic reactivation (Schaal et al., 2015).

On the contrary, in a randomized, counterbalanced and crossover design, 14 habituated English Premier League academy soccer players, Russell and colleagues found that a single WBC session (−135°C, 2 min), performed within 20 min after a repeated sprint exercise (15 × 30 m), increased salivary testosterone, yet had no effect on cortisol, blood lactate, and CK nor on performance (peak power output), recovery, and soreness perceptions (Russell et al., 2017).

It is worth noting that, most of the data reviewed had been obtained from athletes off season or during preparation training phase. It mostly come from endurance athletes or from athletes with a dominating aerobic metabolism. Limited data exist on the conjunction of resistance training and WBC.

In summary, the following reports have been made about the WBC treatment:

- WBC could limit the release of intracellular enzymes, but only after a prolonged cycle of consecutive sessions.
- WBC-associated improvements in muscular tiredness, pain, and well-being after strenuous exercise have been reported in the majority, but not all, of the reviewed studies.
- WBC-mediated enhancement of muscular recovery depends on the limitation of the exercise-induced inflammatory response.

## Performance recovery

Performance recovery using different cooling methods, especially CWI and contrast water immersion, has been extensively studied so far. Their average effect on recovery of trained athletes is rather limited, as reported in a recent review, but under appropriate conditions (whole-body cooling, recovery from sprint exercise) post-exercise cooling has positive effects even for elite athletes (Poppendieck et al., 2013).

Positive effects induced by WBC after 96 h were reported in 18 physically active subjects, who performed a single maximal eccentric contractions of the left knee extensors, through two WBC sessions (−110°C) 24 and 48 h after exercise. The effects were negative at 24 and 48 h post-exercise (Costello et al., 2012a). Positive effects were also reported 24 and 48 h after the treatment in nine runners completing a simulated 48-min trail run, submitted to three WBC sessions, immediately after the exercise as well as 1 and 2 days after (Hausswirth et al., 2011).

Eleven endurance athletes were tested twice in a randomized crossover design with 5 × 5 min of high intensity running followed by 1 h of passive recovery, including either WBC (−110°C, 3 min) or a 3 min walk. Time-to-exhaustion difference between a ramp-test protocol before running and 1 h post-recovery was lower in WBC-treated subjects. WBC improves acute recovery during high-intensity intermittent exercise in thermoneutral conditions. This could be induced by enhanced oxygenation of the working muscles as well as by reduction of cardiovascular strain and increased work economy at submaximal intensities (Krüger et al., 2015). In addition to beneficial effects on inflammation and muscle damage, WBC induces peripheral vasoconstriction, which improves muscle oxygenation (Hornery et al., 2005), lowers submaximal heart rate and increases stroke volume (Zalewski et al., 2014), stimulates autonomic nervous parasympathetic activity and increases norepinephrine (Hausswirth et al., 2013). These effects favor post-exercise recovery and induce analgesia (Krüger et al., 2015).

Although these evidences, a recent meta-analysis by Bleakley et al., based on a small number of randomized studies, highlighted that WBC sustains improvements in subjective recovery and muscle soreness following metabolic or mechanical overload, but little benefit toward functional recovery (Bleakley et al., 2014). The authors concluded that, until further researches will be available, less expensive cooling modality (local ice-pack, cold water immersion) would be used in order to gain the same physiological and clinical effects to WBC.

## Exposure

### Time

Three-minute WBC exposure significantly differ from a 1–2-min exposure. Blood volume decreased within vastus lateralis and gastrocnemius occurred 0–5 min after WBC in 14 professional rugby players. Oxyhemoglobin and deoxyhemoglobin increased in 15 min post-WBC, reaching baseline values indicative of venous pooling. Extreme cold induces vasodilation after constriction in very short time. Gastrocnemius is more susceptible to pooling at all exposure times than vastus lateralis. Two-minute WBC exposure causes changes in core and T_sk_, tissue oxygenation in vastus lateralis, and gastrocnemius and thermal sensation. The optimum exposure time is 30 s at −60°C followed by 2 min WBC at −135°C (Selfe et al., 2014).

It is also crucial to keep a constant temperature between two consecutive treatments. Door opening and subject permanence within a chamber increase temperature and reduce therapeutic effectiveness, particularly for electrical cryochambers, but also for liquid nitrogen-cooled chambers. A 2 min wait between two consecutive treatments would allow temperature recovery to therapeutic levels.

### Sessions

The number of sessions is crucial for WBC effectiveness, as previously discussed. A recent Cochrane review, reporting on the absence of beneficial effects of WBC on prevention and treatment of muscle soreness in athletes, involves on only four papers. One out of these four papers talked about six treatments in cryocabin, the other two investigated the effects of a single treatment in a cryochamber and the final one reported the effects of only three treatments in a cryochamber (Costello et al., 2015). A single session is probably not sufficient to exert any significant effect. Twenty consecutive sessions should be a minimum for effectiveness evaluation; 30 sessions should be the optimum, because a complete hematological and immunological recovery after the initial response is possible (Szygula et al., 2014). Studies evaluating long-term WBC treatment are not easily performable in professional athletes during competitive seasons, but they could be proposed during training and summer camps. Although offseason injuries are rarer than contusions incurred during competitions, it is important to note that standardization of exercise and training offseason is more easily achievable.

Furthermore, randomization is very difficult, if not impossible, to be proposed to elite athletes, and professional teams: the treatment is proposed to improve recovery or to prevent injuries, thus, it should not be limited to a subgroup of athletes. On the other hand, when WBC is used for accelerating recovery from trauma/injury, only injured athletes are treated. Crossover studies could be more easily performed during training camps (but not during competitive season), but they would be only devoted to physiological modifications and not to recovery.

Different, and sometime discrepant results presented in current literature could be attributable to different levels of subjects ranging from “physically active” to “elite” to “national/international selection.” A stratification of WBC effects should be evoked for different subjects, because of different adaptation to effort, recovery capacity/velocity, and energy metabolism.

## Conclusions

Based on the findings here collected, the majority of evidence supports effectiveness of WBC in relieving symptomatology of the whole set of inflammatory conditions that could affect an athlete. A small number of studies that did not report any positive effects should, however, not be neglected. The same applies to improvement of post-exercise recovery, and noteworthy, to limiting or even preventing EIMD. The perception of WBC is changing from a conventionally intended symptomatic therapy to a stimulating treatment able to enhance the anti-inflammatory and -oxidant barriers and to counteract harmful stimuli. Importantly, cooling effectiveness depends on the percentage of fat mass of a subject and the starting fitness level. These results, combined with evidence that WBC somehow mimics exercise, at least in its ability to induce a pulsatile expression of myokines (IL-6, irisin), open another window of possible therapeutic strategies for obesity and type 2 diabetes.

As above highlighted, some of the applied WBC protocols have been ineffective in inducing appreciable modifications of certain biochemical parameters. However, in these cases, the final clinical output (in a subjective assessment: in terms of pain, soreness, stress, and recovery) was significantly improved even when compared to other recovery strategies.

WBC, used either as a therapy or stimulation, is a medical treatment and as such it has contraindications and standard safety procedures. The undeniable risks for the users can be rendered negligible if all the procedures are conducted following precise rules under supervision of highly-skilled personnel. If these procedures are carefully followed, WBC is absolutely safe.

The scientific debate on WBC, often shaded by non-scientific discussions hold in newspapers and web dictated by curiosity or accidents (recent incident in a non-controlled cryocabin), needs consensus and international cooperation for building up wide and controlled studies.

## Author contributions

GL and EZ: conception and design, data acquisition; drafting paper; final approval; agreement for all the aspects of the work. GB: conception and design, data acquisition; critical revision; final approval; agreement for all the aspects of the work.

## Funding

This work has been funded by an unrestricted grant from the Italian Ministry of Health and grant from the Polish Ministry of Science and Higher Education No. 0026/RS3/2015/53.

### Conflict of interest statement

The authors declare that the research was conducted in the absence of any commercial or financial relationships that could be construed as a potential conflict of interest.
